# Anthropometry at birth and at age of routine vaccination to predict mortality in the first year of life: A birth cohort study in BukinaFaso

**DOI:** 10.1371/journal.pone.0213523

**Published:** 2019-03-28

**Authors:** Martha Mwangome, Moses Ngari, Paluku Bwahere, Patrick Kabore, Marie McGrath, Marko Kerac, James A. Berkley

**Affiliations:** 1 KEMRI/Wellcome Trust Research Programme, Kilifi, Kenya; 2 The Childhood Acute Illness and Nutrition Network, Nairobi, Kenya; 3 School of Public Health, Center of Research in Epidemiology Biostatistics and Clinical Research, Université Libre de Bruxelles, Brussels, Belgium; 4 Valid International, Oxford, United Kingdom; 5 Africa Regional office, World Health Organisation, Brazzaville, Republic of Congo; 6 Emergency Nutrition Network, Oxford, United Kingdom; 7 Faculty of Epidemiology and Population Health, London School of Hygiene and Tropical Medicine (LSHTM), London, United Kingdom; 8 Centre for Clinical Vaccinology & Tropical Medicine, University of Oxford, Oxford, United Kingdom; Centre Hospitalier Universitaire Vaudois, FRANCE

## Abstract

**Background:**

The World Health Organization currently defines severe acute malnutrition (SAM) in infants aged under 6 months of age using weight-for-length Z score (WLZ). Given widespread use of mid-upper arm circumference (MUAC) for identifying SAM in older children and weight-for-age (WAZ) for growth monitoring, there is increasing debate about the optimal anthropometric criteria to best identify infants u6m at-risk of mortality.

**Objective:**

To determine the discriminatory value for mortality during the first 12 months of life of anthropometry taken at birth and at age two months (approximate age of routine vaccination).

**Design:**

Data were analyzed from a birth cohort recruited between April and December of 2004 at four health facilities within Bansalogho District in Burkina Faso. Infants were followed up for 12 months. Mortality risks were estimated using hazards ratios (HR). Discriminatory value was assessed using receiver operating characteristic curves.

**Results:**

Of 1,103 infants, 227 (21%) were low birthweight (LBW). During 12 months, 86 (7.8%) infants died; 38 (44%) among the LBW group. At birth, MUAC<9.0cm, weight<2.5kg, length<44.2cm and incalculable WLZ were associated with mortality. Sixty (70%) deaths occurred after the age of two months; 26 (43%) among LBW infants. At age two months, any MUAC <11.5cm, weight <3.8kg (WAZ<-3) and length <52.4cm (LAZ<-3) were associated with risk of mortality. WLZ was not associated with mortality at any threshold.

Birth weight did not modify the effect of the association between month two MUAC and one-year mortality (P = 0.33).

**Conclusion:**

Infants at heightened risk of mortality and are better identified during early infancy by MUAC or WFA than by WLZ. LBW infants with low anthropometry at the age of routine immunizations remain at elevated risk than normal birth weight (NBW)infants and require intervention. Effectiveness, cost-effectiveness and coverage of applying proposed thresholds should be investigated as a priority to inform policy and practice.

## Introduction

Undernutrition in early infancy has important consequences for survival, long-term health and neurodevelopment. It is estimated that in low and middle income countries (LMICs), 8.5 million infants aged below 6 months (u6m) are wasted (weight for length Z score (WLZ) < -2)[1]. Low-birth-weight (LBW) arising from prematurity or being small-for-gestational age; infections; disability; or sub-optimal breastfeeding (late initiation, non-exclusive) are risk factors for being classified as being malnourished among infantsu6m [2–5]. Infants born LBW are likely to remain smaller and be susceptible to illness, neurodevelopmental problems and later chronic non-communicable diseases [6].

The diagnosis of severe acute malnutrition (SAM) among infants u6m is currently based on WLZ, using the same threshold of <-3SD applied for older children [7]. However, there are concerns about the reliability and accuracy of length measurement in early infancy [8, 9]. Additionally, for infants shorter than 45cm, WLZ cannot be calculated using the 2006 WHO growth standards [10]. Mid-upper arm circumference (MUAC) has shown potential in identifying vulnerable infants u6m at risk of subsequent mortality. In a recent review, MUAC and weight-for-age (WAZ) were rated the best indicators of acute malnutrition in infants u6m. Specifically, MUAC was reported to be simple, acceptable, affordable, objective, age independent, reliably and accurately measured and a better predictor of mortality than WLZ [11]. In a community cohort in rural Gambia [12] and in a hospitalized and post-discharge cohort in rural Kenya [13], MUAC taken around the time of infant immunizations (6 to 14 weeks after birth) better discriminated subsequent mortality than WLZ. Current evidence suggests that a MUAC criterion of <11.0cm could be applied to identify SAM among infants aged 2 to 6 months [13]. However, this threshold needs further validation before it can be applied to policy. In this same study, a large proportion of infants u6m classified as acutely malnourished were born LBW and were observed to have a considerably higher mortality risk than normal birth weight (NBW) infants [13]. Importantly, LBW does not distinguish preterm birth from intrauterine growth retardation. A LBW infant who is small but growing parallel to centiles could therefore later be classified as ‘malnourished’. As studies have usually excluded neonates (<28 days), the long-term predictive value for mortality of birth MUAC is unknown. Additionally, non-weight based correlates of LBW may be of value for assessing risks in non-facility births. MUAC has consistently reported better performance than other measures to identify LBW at birth [14–17]. However, proposed MUAC cut-offs to discriminate LBW differ between studies, ranging from 9.0cm to 10.0cm, without consensus. This analysis aimed to determine the discriminatory value of MUAC taken at birth and at the age of routine vaccination (2 months) for subsequent mortality up to 12 months of age. Secondarily, the analysis aimed to determine MUAC’s value in identifying LBW at birth. To do this, we performed a secondary analysis of data from a birth cohort in Bukina Faso that aimed to compare survival of LBW and NBW infants [18, 19].

## Methods

### Study site

Burkina Faso is a resource-poor land-locked country in West Africa. This analysis utilized data from a birth cohort within an urban area of Barsalogho Health District, part of the Kaya Health Region in Central North Burkina Faso. The District is located at the edge of the Sahel with a population of predominantly subsistence farmers. Data were collected between April and December of 2004 in four different health centers including Barsalogho, Basma, Dablo, and Foubé.

### Study population and design

This was a secondary analysis of a prospective birth cohort. The parent study recruited pregnant women attending scheduled antenatal care (ANC) in their 3^rd^ trimester. Only women who consented were included in the study. This analysis, included data from all live hospital and home births within the cohort who were followed up for one year, loss to follow up or death. The primary outcome of this analysis was death within 12 months from the date of birth. In this study, LBW was defined as birth weight <2.5kg, and prematurity was defined as estimated gestational age <37 weeks. The exposures examined at birth were birth weight (kg), MUAC (cm) and length (cm); while exposures examined at month two were weight (kg), MUAC (cm), length (cm), WLZ, concurrent wasted and underweight, concurrent wasted and stunted, and concurrent stunted, wasted and underweight.

### Data sources/measurements

Parental demographic characteristics were collected at birth. Infant anthropometry was measured within 2 hours after a hospital birth, and within 48 hours for a home birth, then monthly for twelve months. MUAC in cm was measured using a 1 mm graded non-elastic measuring tape, weight was measured within 10 grams using a SECA baby scale while length was measured using a 1cm graded measuring stick. Deaths were recorded at every visit, ascertained using hospital documents for hospital deaths and verbal autopsy for community deaths.

### Study size

With the fixed sample of 1103 infants and 86 deaths after 12 months of age, there was >90% power to detect a hazard ratio >3.0 of association between birth MUAC cut-off of 9.0cm and 12 months’ mortality with 0.05 level of precision.

### Statistical analysis

Weight-for-age (WAZ), weight-for-length (WLZ) and length for age (LAZ) at birth and month were calculated two using the 2006 WHO growth references that are not adjusted for gestational age, which could not be precisely ascertained, as in routine practice. Stunted, wasted and underweight were defined as LAZ<-2, WLZ<-2 and WAZ<-2 respectively. Concurrently wasted and stunted were defined as infants with WLZ<-2 and LAZ<-2, concurrent wasted and underweight as WLZ<-2 and WAZ<-2, and concurrent stunted, wasted & underweight as LAZ<-2, WLZ<-2 and WAZ<-2. Non-malnourished infants were defined as those with Z scores ≥-2 for these parameters. Missing anthropometric data was analyzed as a separate category. Infant’s anthropometry at birth, at month two and at month twelve were summarized using means and standard deviations and compared children with LBW and NBW (birth weight ≥2.5kg) using z-test with α<0.05 considered as the statistical level of significance.

In this cohort, because anthropometric measures for all infants were taken at the same intervals i.e. at birth, age month 1, age month 2 and so on up to month 12, the use of age standardized Z scores such as WAZ and LAZ was not seen to add any practical benefit. Hence, absolute weight and length cut-offs corresponding to <-2 and <-3 WAZ and LAZ standards at each month of age were used instead [10]. MUAC cut-offs used in this analysis were derived from previous studies on the utility of MUAC at birth [15, 16] and post-neonatal MUAC up to 6 months of age [12, 13].

To estimate discrimination of mortality risks by anthropometric measures taken at birth and at month 2 for mortality up to 12 months of age, the area under receiver operating characteristics curves (AUC) was calculated. To test for an association of birth and month 2 anthropometry with one-year mortality, single event survival analysis was used. Time at risk was defined as time from birth or month two to date of death, lost-to-follow-up or last day of follow-up (one year after birth) for mortality after birth and month two analysis respectively. Cox proportional hazards regression was used to determine the association between anthropometry and mortality, and reported hazard ratios (HR) with 95% confidence intervals. The Schoenfeld residuals test was used to test for proportional hazard assumption. For univariable regression analysis, each anthropometric indicator was analyzed separately as the only independent variable in the model and for the multivariable regression analysis, *a priori* confounders (gender, recruitment facility and month of birth) selected during the development of the secondary data analysis were added. There were 14 pairs of twins recruited, therefore twin clustering was adjusted for in the regression models. Survival curves were fitted using Kaplan-Meier method and log-rank test used to compare survival distributions between different groups. To test for effect modification of birth weight on the association between birth MUAC (cm) and one-year mortality, likelihood-ratio tests comparing models with and without interaction terms were used. Premature and twin infants are known to have a higher risk of mortality and different growth trajectory, therefore, a sub-analysis to evaluate the effect of anthropometry on risk of death on infants who are not known to be born premature or twins was performed.

To identify a MUAC proxy for LBW, two approaches were used; A) assessed a linear association using the Pearson correlation coefficient and plotted a scatter plot with the estimated line of best linear fit and checked the MUAC cut-off where a reference line of birth weight of 2.5kg intersected with the best line fit; and B) computed sensitivity and specificity at different MUAC cut-offs, and determined the statistically optimal MUAC cut-off using the square of distance method [20].

Stata version 15.1 (StataCorp, College Station, TX, USA) was used to perform all the statistical analysis.

### Ethical considerations

Approval for data collection was granted by the Ministry of Health of Burkina Faso in 2003 (approval number: 1014) in accordance with national procedure. All data were anonymized before being shared for this analysis.

## Results

### Cohort characteristics

A total of 1,103 infants u6m (S1 Fig) recruited from four health centres, Basma 416 (38%); Bansalongho: 320 (29%); Dablo: 286 (26%); and Foube: 81 (7.3%) were included in this analysis; 533 (48%) were female. Mean (sd) birth weight, length and MUAC were: 2.83 (0.5) kg, 48.9 (2.6) cm and 10.2 (1.1) cm respectively. Two hundred and twenty-seven (21%) infants were born LBW (48 infants (21%) of whom were reported premature). The distribution of LBW did not differ significantly by calendar month (p = 0.12). A total of 492/1,103 (45%) infants were born at health facilities (HC), 432 (39%) at home with the assistance of Community Birth Attendants (CBA) and 179 (16%) at home in absence of trained birth attendants. There were thirty-nine (39) reported individual twins within the study however only 14 pairs (28 infants) were recruited into the study, suggesting non-survival of the other twin. Of the 39 reported twins, 29/227 (13% of total LBW) were LBW and 10/876 (1.1%) were NBW, P<0.001. There were 62 infants reported to have been born prematurely (5.6% of all infants enrolled; 27% of LBW infants) with median gestational age of 35 months (IQR 33 to 36 months). The proportion of preterm & LBW infants did not differ significantly by calendar month (P = 0.25, P = 0.12 respectively). Of the 62 preterm infants, 35 (56%) were born at a health center, 14 (23%) were delivered at home by CBA while 13 (21%) were unassisted home deliveries. Of the 62 preterm infants, 12 (19.4%) were twins.

Mothers’ median (IQR) age was 24 (20–30) years; mothers of infants born with LBW were younger (21 (IQR 19–27) years) than the mothers of infants born with NBW (25 (IQR 20–30) years; p = 0.001). The median (IQR) height of mothers was 163 (159–168) cm. Eight hundred and fifty-four (77%) mothers were illiterate and 822 (75%) were Muslim. The median (IQR) age ofthe infants’ fathers was 39 (31–49) years, and 568 (53%) fathers were illiterate. The mean (sd) father’s BMI was 21.5 (2.6).

Fifty-nine (5.3%) infants had incalculable WLZ at birth as their birth length was below 45cm. The risk of death associated with missing birth WLZ was analyzed separately. One hundred and forty-seven infants had missing month 2 anthropometry as they failed to attend their month 2 visit. Two infants measured at month two had length <45cm and therefore has incalculable WLZ.

83/1103 (7.5%) infants were lost-to-follow-up after median 210 (IQR 93–304) days. There were no significant differences in birth weight (P = 0.35), length (P = 0.50) and MUAC (P = 0.24) between the infants lost-to-follow-up and those not lost-to-follow-up.

### The Prevalence of undernutrition

A total of 661 (60%) infants were not stunted, wasted or underweight at birth. The prevalence of being wasted among all infants at birth was 30% by WLZ<-2 and 7.2% by MUAC<9.0cm, 10% were stunted (LAZ<-2) and 17% were underweight (WAZ<-2). (Table 1). Wasted, stunted, underweight and all concurrent forms of undernutrition at birth, two months and twelve months were more prevalent among infants born LBW compared to those born NBW (all p<0.001). Among all infants, being wasted was more common than being stunted and generally declined over time. The proportion of infants who were stunted doubled between birth and 2 months with little change thereafter. The proportion of concurrently wasted and stunted infants increased over time among all the groups (Table 1). Infants born with LBW had lower weight than those born with NBW at both months two (p<0.001) and month twelve (p<0.001) (S1 Table).

**Table 1:** Anthropometric categories at birth, two and twelve months of age.

	At birth (N = 1103) N (%)	At month 2 (N = 927) N (%)	At month 12 (N = 941) N (%)
**All infants (N = 1103)**			
No stunted, no wasted & no underweight	661 (60)	577 (62)	551 (50)
Stunted (HAZ <-2)	114 (10)	187 (20)	167 (18)
Wasted (WLZ <-2)*	327 (30)	152 (16)	260 (28)
Severely wasted (WLZ <-3)*	146 (14)	65 (7.0)	99 (11)
MUAC <11.5cm	955 (87)	160 (17)	38 (4.0)
MUAC <11.0cm	735 (67)	63 (6.8)	12 (1.3)
MUAC <10.0cm	327 (30)	17 (1.8)	2 (0.2)
MUAC <9.0cm	79 (7.2)	6 (0.6)	0
Underweight (WAZ <-2)	185 (17)	148 (16)	307 (33)
Concurrent stunted & wasted	13 (1.2)	9 (1.0)	66 (6.0)
Concurrent wasted & underweight	107 (9.7)	51 (5.5)	220 (20)
Concurrent stunted & underweight	77 (7.0)	84 (9.0)	124 (11)
Concurrent stunted, wasted & underweight	13 (1.2)	10 (1.1)	66 (6.0)
**NBW infants (N = 876)**	N = 876	N = 740	N = 762
No stunted, wasted and underweight	645 (74)	519 (70)	489 (56)
Stunted (HAZ<-2)	32 (3.7)	108 (15)	98 (13)
Wasted (WLZ<-2)	199 (23)	104 (14)	194 (25)
MUAC <11.5cm	731 (83)	81 (11)	22 (2.9)
MUAC <11.0cm	515 (59)	21 (2.8)	6 (0.8)
Underweight (WAZ <-2)	0	54 (7.3)	205 (27)
Concurrent stunted & wasted	0	2 (0.3)	38 (4.3)
Concurrent wasted & underweight	0	23 (3.1)	160 (18)
Concurrent stunted & underweight	0	22 (3.0)	64 (7.3)
Concurrent stunted, wasted & underweight	0	2 (0.3)	38 (4.3)
**LBW infants (N = 227)**	N = 227	N = 187	N = 179
No stunted, wasted and underweight	16 (7.1)	58 (31)	62 (27)
Stunted (HAZ <-2)	82 (36)	79 (42)	69 (39)
Wasted (WLZ <-2)	128 (56)	48 (26)	66 (37)
MUAC <11.5cm	224 (99)	79 (42)	16 (8.9)
MUAC <11.0cm	220 (97)	42 (22)	6 (3.4)
Underweight (WAZ <-2)	185 (82)	94 (50)	102 (57)
Concurrent stunted & wasted	13 (5.7)	8 (4.3)	28 (12)
Concurrent wasted & underweight	107 (47)	29 (16)	60 (26)
Concurrent stunted & underweight	77 (34)	63 (34)	60 (26)
Concurrent stunted, wasted & underweight	13 (5.7)	8 (4.3)	28 (12)

*59 children missing birth WLZ because birth height <45cm,

MUAC-mid-upper arm circumference, WLZ-Weight-for-length z-score, WAZ-Weight-for-age z-score, HAZ-Length-for-age z-score

### Birth anthropometry to discriminate subsequent mortality

**Fig 1:**
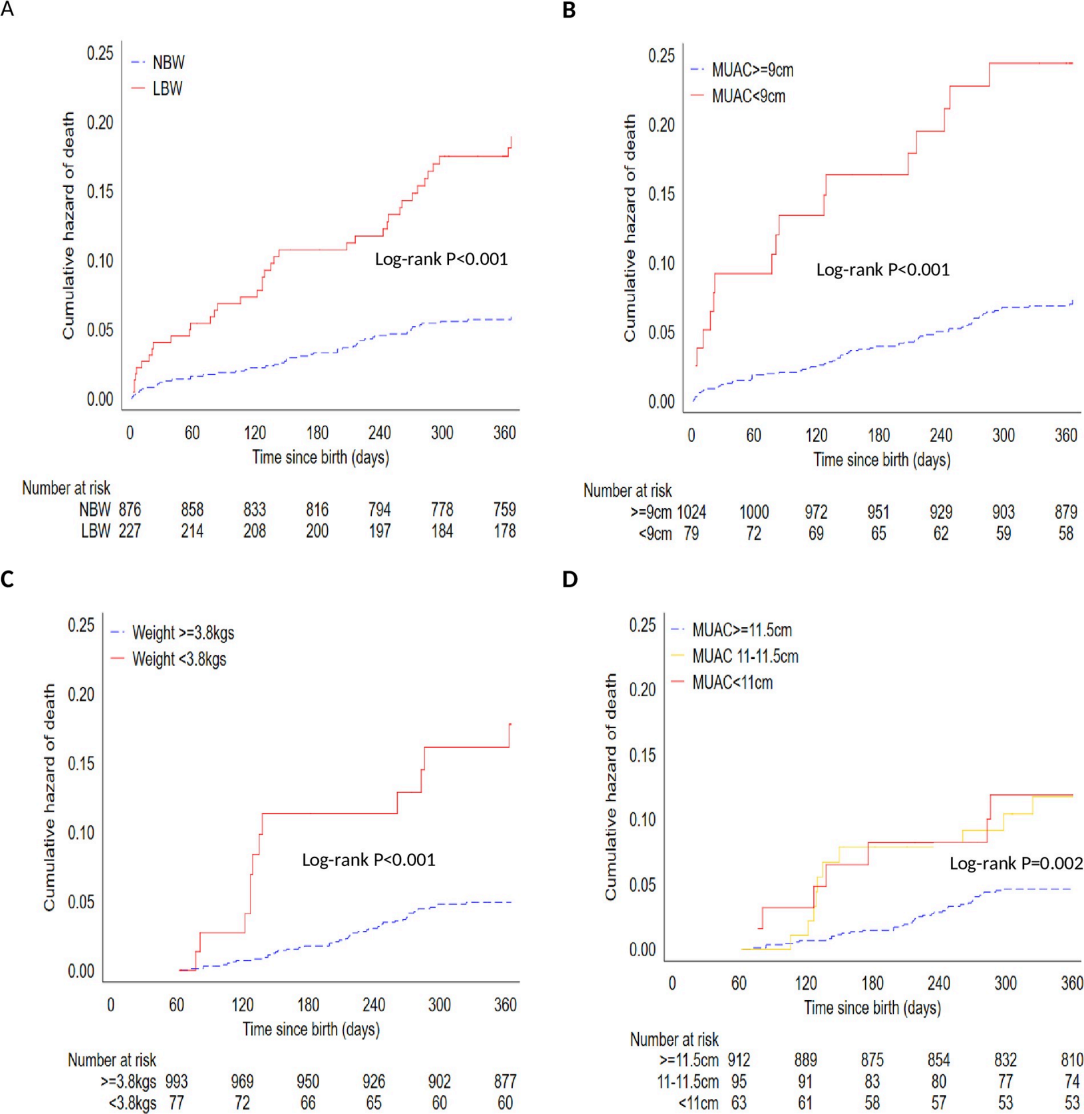
Nelson-Aalen cumulative hazard of one-year mortality. A: stratified by LBW, B; stratified by Birth MUAC<9.0 cm, C; stratified by month two weight<3.8kg and D; stratified by month two MUAC.

Within the twelve months after birth, 86/1,103 (7.8%, 95% CI 6.3–9.5%) infants died during 1,015 child-years of observation (cyo), mortality rate 84.8 (95% CI 68.6–104.7) deaths per 1000 cyo. A total of 26/86 (30%), 31/86 (36%) and 51/86 (59%) deaths occurred by two, three and six months after birth (**SI Fig**). There were 38/227 (17%) deaths amongst infants born with LBW during the 12 months of follow-up during 199.8 cyo; 190.1 (95% CI 138.4, 261.3) per 1000 cyo; and amongst infants born NBW, 48/876 (5.5%) deaths occurred during 815 cyo; 58.9 (95% CI 44.4, 78.2) deaths per 1000 cyo (P<0.001). The hazards ratio for death associated with LBW, adjusted for gender, month and facility of recruitment, was 3.12 (95% CI 2.01, 4.88) (Fig 1A). However, the risk of death among LBW infants increased compared to NBW infants over time (Fig 1A). The proportional hazard assumption was not violated (P = 0.88).

The AUCs for birth anthropometry for discriminating mortality were greater than 0.5 except for WLZ (Table 2). Missing WLZ at birth was more strongly associated with mortality; HR 3.57 (CI 1.89, 6.75) than having WLZ<-3 at birth; HR 1.89 (CI 1.02, 3.48).

**Table 2:** Birth anthropometry and mortality to 12 months of age.

			Crude (95% CI)		Adjusted (95% CI)^#^	
	N (1103)	Deaths N (%)	HR (95% CI)	P value	HR (95% CI)	P value
**Birth anthropometrics (Deaths, N = 86)**						
**Mid-upper arm circumference (MUAC-cm)**						
MUAC ≥10.0cm	776	51 (6.6)	Reference		Reference	
MUAC 9.0 to 10.0cm	248	18 (7.3)	1.08 (0.63, 1.85)	0.78	1.25 (0.71, 2.19)	0.44
MUAC <9.0cm	79	17 (22)	3.34 (1.87, 5.97)	<0.001	3.97 (2.18, 7.22)	<0.001
**Weight (kg)**						
Weight ≥2.5kg (WAZ ≥-2)	876	48 (5.5)	Reference		Reference	
Weight 2.0 to 2.5kg (WAZ -2 to -3)	179	26 (15)	2.66 (1.65, 4.30)	<0.001	2.75 (1.72, 4.41)	<0.001
Weight <2.0kg (WAZ <-3)	48	12 (25)	4.72 (2.40, 9.27)	<0.001	4.52 (2.25, 9.08)	<0.001
**Length (cm)**						
Length ≥46.1cm (LAZ ≥-2)	955	62 (6.5)	Reference		Reference	
Length 44.2 to 46.1cm (LAZ -2 to -3)	93	10 (11)	1.68 (0.85, 3.33)	0.14	1.70 (0.87, 3.36)	0.12
Length <44.2cm (LAZ <-3)	55	14 (25)	4.01 (2.14, 7.49)	<0.001	3.93 (2.10, 7.38)	<0.001
**Weight-for-length z-score (WLZ)**						
WLZ ≥-2	717	48 (6.7)	Reference		Reference	
WLZ -3 to -2	181	7 (3.9)	0.56 (0.26, 1.23)	0.15	0.58 (0.26, 1.28)	0.18
WLZ <-3	146	17 (12)	1.72 (0.99, 2.98)	0.05	1.89 (1.02, 3.48)	0.04
Missing WLZ	59	14 (24)	3.56 (1.90, 6.69)	<0.001	3.57 (1.89, 6.75)	<0.001
**Birth anthropometry (Continuous) AUCs**	N	Deaths				
MUAC (cm)	1103	86	0.58 (0.51, 0.65)			
Weight (kg)	1103	86	0.60 (0.53, 0.68)			
Length (cm)	1103	86	0.61 (0.54, 0.68)			
Weight-for-length z-score (WLZ)*	1044	72	0.51 (0.44, 0.59)			

AUC-area under receiver operating characteristic, MUAC-mid-upper arm circumference,

^#^-adjusted for gender, facility of recruitment and month of birth,

*59 missing WLZ because their birth lengths<45cm.

A birth MUAC of <9.0cm was associated with elevated hazard of death; HR 3.97 (95% CI 2.18, 7.22). Birth MUAC of between 9.0 to 10.0cm was not associated with mortality compared to ≥10.0cm; HR 1.25 (95% CI 0.71, 2.19) (Table 2 **and** Fig 1B).

Birth weight of between 2.0–2.5kg was associated with risk of death; HR 2.75 (95% CI 1.72, 4.41) compared to birth weight of ≥2.5kg. Birth length <42.2cm (denoting birth LAZ<-3) was associated with risk of death; HR 3.93 (95% CI 2.10, 7.38 compared to birth length ≥44.2cm. Birth length of <46.1cm (denoting LAZ<-2,) was associated with 2.71 (95% CI 1.60,4.60) compared to birth length of ≥ 46.1cm). (Table 2).

There was evidence that birth weight modified the effect of the association between birth MUAC and one-year mortality (P = 0.001). When infants were stratified by LBW, birth MUAC was associated with one-year mortality amongst LBW infants and not NBW infants (S2 Table).

In the sub-analysis, of the 89 infants born either premature or a twin, 20/89 (23%) died in the first year of life; 270.3 (95% CI 174.4, 419.0) deaths per 1000 cyo. The adjusted (birth health facility, gender and month of birth) risk of mortality associated with being premature or twin was HR 3.83 (95% CI 2.30 to 6.38). Excluding known premature births and twins, birth MUAC thresholds below 9.0cm and birth length <44.2cm (LAZ <-3) were not associated with mortality; HR 1.03 (95% CI 0.25 to 4.31) and HR 2.10 (95% CI 0.65 to 6.81) respectively. However, birth weight below 2.5kg was associated; HR 2.32 (95% CI 1.34 to 4.03) (S3 Table).

### Birth MUAC to identify LBW

**Fig 2:**
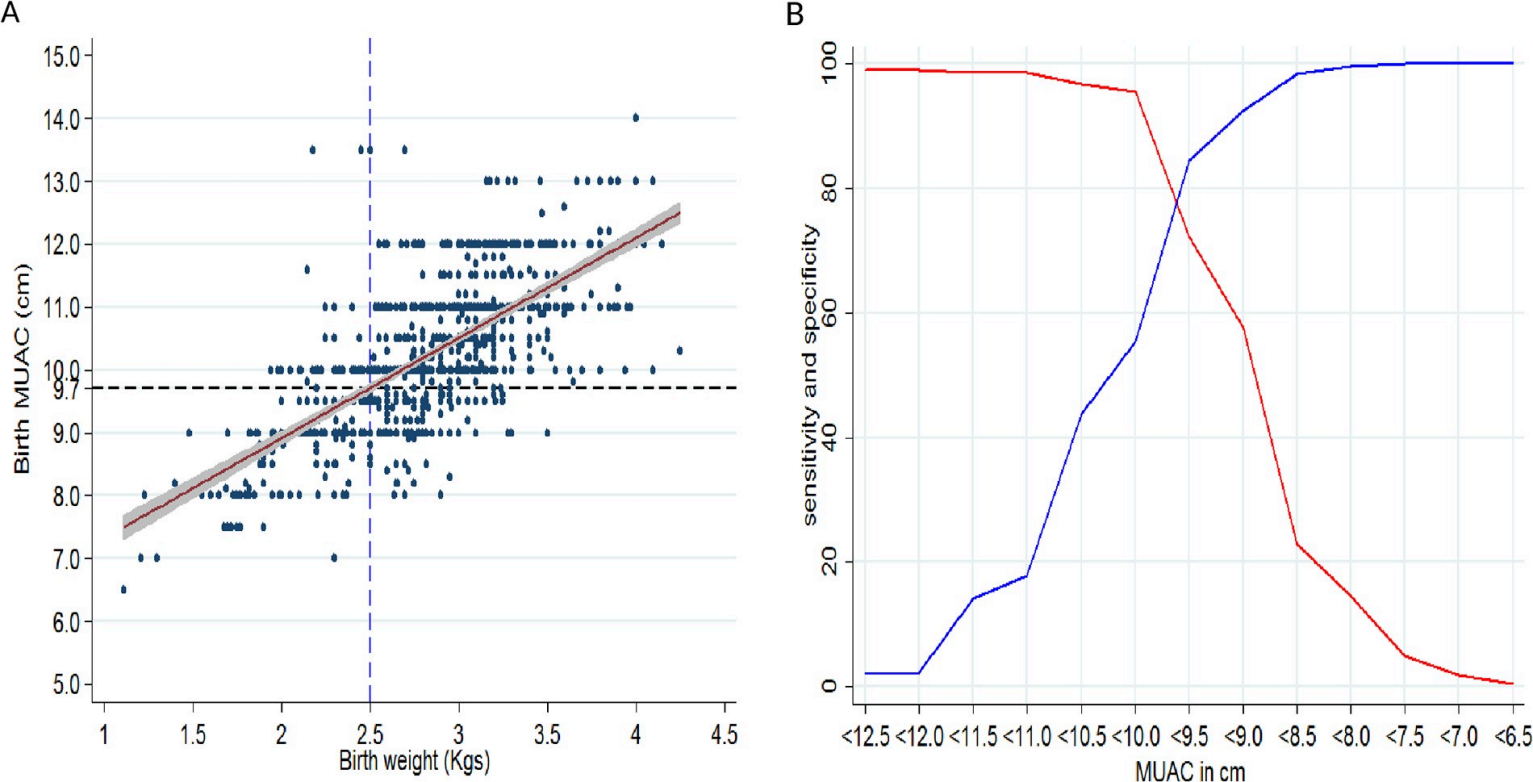
A) A scatter plot of birth MUAC (cm) and birth weight (kg); B) A plot of diagnostic accuracy of different birth MUAC cut offs for LBW (weight <2.5kg).

Birth MUAC had a strong positive correlation with birth weight; Pearson correlation coefficient was 0.68 (Fig 2A).

The linear best fit, adjusted for gender, was; birth MUAC (cm) = 5.72 + 1.60 (95% CI 1.49, 1.70) (birth weight in kg). The predicted birth MUAC by the linear regression equation was 9.7cm for birth weight of 2.5kg as shown by intersection of the two reference lines on Fig 2A. From the plot of sensitivity and specificity against different MUAC cut-offs the statistically optimal birth MUAC cut-off was 9.7cm as shown by the intersection of the sensitivity and specificity lines (Fig 2B). Within this cohort, applying the birth MUAC cut-off of <9.7cm, identified 164 of the 227 LBW infants with a sensitivity of 72.3% and specificity of 84.6%.

Overall, the MUAC cut-off of <9.7 cm at birth that best discriminated LBW was associated with a hazard ratio (adjusted for gender, month of birth and facility of delivery) for death of 1.98 (95% CI 1.23, 3.18, P = 0.005) compared to infants ≥9.7cm (S2 Fig).

### Anthropometry at the age of vaccination to predict mortality

A total of 60/86 (70%) deaths occurred after the first two months of life, during 837 cyo; 71.7 (95% CI 55.7, 92.4) deaths per 1000 cyo. Of these 60 deaths, 26/60 (43%) occurred among LBW infants; 158.6 (95% CI 108.0, 232.9) per 1000 cyo and amongst the infants born with NBW 34/60 (57.0%) deaths occurred; 50.5 (95% CI 36.1, 70.7) deaths per 1000 cyo; adjusted hazards ratio 2.89 (95% 1.69, 4.95) (Table 3).

**Table 3:** Month two anthropometry and mortality to 12 months of age.

			Crude (95% CI)		Adjusted (95% CI)^#^	
	N (927)	Deaths N (%)	HR (95% CI)	P value	HR (95% CI)	P value
**Month 2 Anthropometry (Death, N = 60)**						
**Mid-upper arm circumference (MUAC-cm)**						
MUAC ≥11.5cm	765	27 (3.5)	Reference		Reference	
MUAC 11.0 to 11.5cm	95	10 (11)	3.18 (1.54, 6.55)	0.002	3.43 (1.58, 7.46)	0.002
MUAC <11.0cm	63	8 (13)	3.14 (1.35, 7.34)	0.008	3.49 (1.44, 8.46)	0.006
Missing MUAC	147	15 (10)	3.13 (1.68, 5.81)	<0.001	2.77 (1.44, 5.31)	0.002
**Weight (kg)**						
Weight ≥4.3kg (WAZ ≥-2)	733	25 (3.4)	Reference		Reference	
Weight 3.8 to 4.3kg (WAZ -2 to -3)	113	7 (6.2)	1.82 (0.81, 4.11)	0.15	1.78 (0.77, 4.10)	0.18
Weight <3.8kg (WAZ< -3)	77	13 (17)	4.79 (2.31, 9.92)	<0.001	4.68 (2.23, 9.82)	<0.001
Missing weight	147	15 (10)	3.22 (1.71, 6.06)	<0.001	2.82 (1.46, 5.46)	0.002
**Length (cm)**						
Length ≥54.4cm (LAZ ≥-2)	661	25 (3.8)	Reference		Reference	
Length 52.4 to 54.4cm (HAZ -3 to -2)	143	6 (4.2)	1.05 (0.42, 2.62)	0.92	1.10 (0.43, 2.86)	0.84
Length <52.4cm (HAZ <-3)	119	14 (12)	3.06 (1.58, 5.93)	0.001	2.88 (1.41, 5.86)	0.004
Missing length	147	15 (10)	2.90 (1.55, 5.43)	0.001	2.74 (1.40, 5.36)	0.003
**Weight-for-length z-score (WLZ)**						
WLZ ≥-2	770	33 (4.3)	Reference		Reference	
WLZ -3 to -2	87	5 (5.8)	1.25 (0.50, 3.14)	0.63	1.14 (0.44, 2.94)	0.78
WLZ <-3*	64	6 (9.4)	2.13 (0.88, 5.12)	0.09	1.84 (0.75, 4.51)	0.18
Missing WLZ	149	16 (11)	2.73 (1.52, 4.91)	0.001	2.38 (1.28, 4.44)	0.006
**Month 2 anthropometry (Continuous) AUCs**	N	Deaths				
MUAC (cm)	923	45	0.62 (0.52, 0.71)			
Weight (kg)	923	45	0.64 (0.55, 0.74)			
Length (cm)	923	45	0.62 (0.53, 0.71)			
WLZ	921	44	0.56 (0.46, 0.65)			
**Concurrent Undernutrition**						
No stunting, wasting or underweight	575	20 (3.5)	Reference		Reference	
Concurrent stunted & wasted	9	3 (33)	9.68 (3.30, 28.41)	<0.001	9.11 (3.64, 22.79)	<0.001
Concurrent wasted & underweight	51	5 (9.8)	3.03 (1.15, 7.97)	0.03	2.79 (1.05, 7.40)	0.04
Concurrent stunted & underweight	84	12 (14)	4.16 (1.99, 8.68)	<0.001	3.28 (1.57, 6.83)	0.002
**Low birth weight status at birth**						
Birth weight ≥2.5kg	856	34 (4.0)	Reference		Reference	
Birth weight <2.5kg	214	26 (12)	2.85 (1.68, 4.86)	<0.001	2.89 (1.69, 4.95)	<0.001

The crude and adjusted hazard ratio are from the Cox regression model; WAZ-Weight for age z-score, LAZ-Length for age z-score, WLZ- Weight for length z-scores, AUC-area under receiver operating characteristic,

^#^-adjusted for gender, facility of recruitment and month of birth.

*2 missing WLZ because their month 2 lengths<45cm.

At month two, any MUAC thresholds below 11.5cm, weight threshold below 3.8kg (WAZ<-3) and length thresholds below 52.4cm (LAZ<-3) were associated with increased risks of subsequent death (Table 3, Fig 1C and 1D). Notably, low WLZ (<-2 and <-3) thresholds were not associated with risk of subsequent mortality (Table 3). Missing MUAC, weight, length (cm) and WLZ were associated with higher risk of subsequent mortality (Table 3).

At month two, MUAC, weight and length discriminated subsequent mortality as the AUCs and their confidence intervals were greater than 0.5 (Table 3). Importantly, the CIs for the AUC for WLZ included 0.5 suggesting WLZ was not a valid predictor (Table 3). There was no evidence that birth weight modified the effect of the association between month two MUAC and one-year mortality (P = 0.33).

In the sub-analysis excluding premature infants and twins, the association between MUAC threshold below 11.5cm and weight <3.8kg with mortality was maintained; HR 2.85 (95% CI 1.14 to 7.13) and HR 4.18 (95% CI 1.68 to 10.38) respectively (S3 Table). Notably, length and WLZ thresholds were not associated with subsequent mortality (S4 Table).

## Discussion

Anthropometric thresholds for intervention are typically based on their association with the risk of mortality in untreated populations. Prior cohort studies of this kind have usually excluded young infants. As a result, in the absence of evidence, WHO and international recommendations for infants under 6 months have adopted the criteria used for older children [7]. A key question challenging the simple use of anthropometry in infancy is whether infants born LBW and growing normally may be detected as ‘malnourished’ when in fact they are at low risk and not requiring specific intervention.

We found that at birth and at 2 months of age, current weight, birth weight, MUAC and length discriminated subsequent mortality. However, WLZ, the current diagnostic criterion for acute malnutrition did not; at birth likely because many WLZ values were incalculable using 2006 growth standards. Rather than being associated with lower risk, LBW modified the effect of birth anthropometry on the risk of death, and did not alter risk at any given MUAC or WAZ taken at 2 months and remained a strong risk factor for death throughout the first year of life.

### Undernutrition in early infancy

Our results indicate a high prevalence of low anthropometry at birth; 21% of the infants were born LBW, which was higher than the global prevalence of 16% at the time of the study [21]. Assessing birth nutritional status can be challenging. In most settings, WHO 2006 growth references are applied [22], however these references do not account for gestational age [23]. The more recent INTERGROWTH-21^st^ growth curves aim to help resolve this problem by creating separate preterm postnatal growth standards [24, 25]. however, their application require knowledge of gestational age, which in LMIC settings is usually either unknown or inaccurately estimated because of the lack of widespread antenatal ultrasound. For these reasons, our analysis examined the application of absolute birth anthropometric measures with unknown gestational age as these would be the more realistic situation in resource poor settings.

### Predictive value of anthropometry

Ideally, an appropriate anthropometric cut-off should depend on how effective or cost-effective an intervention is when applied using that threshold. However, in the absence of such data, predictive and discriminatory values for mortality are used. From our analysis, birth weight <2.5kg, birth MUAC <9.0cm and birth length <44.2cm were strongly associated with mortality. In most resource poor settings, birth length is not routinely measured due to lack of appropriate equipment (infantometer) and home deliveries. Birth weight is commonly recorded in facilities and our results support that it is a useful marker of survival. Birth MUAC is not routinely measured as consensus on its interpretation is lacking. Among infants u6m, lay health workers measured MUAC more reliably and accurately than they did length [9].

Different anthropometric measures have previously been studied for ability to predict LBW. LBW is an indicator of mortality and disability risks, hence other anthropometric measures aiming to predict LBW become indicators of an indicator of risk (*proxy of proxy*). Our findings suggest that the risk of death associated with birth MUAC<9.0cm was similar to that associated with birth weight<2.5kg, likely because it captured prematurity. However, a MUAC threshold of <9.7cm was statistically optimal to identify LBW. Our results slightly differ from findings for Uganda and Ghana where MUAC<10.1cm and MUAC <9.4cm respectively better predicted LBW [16, 17]. These sites may have differed in the proportion of LBW due to prematurity.

MUAC has the potential to be measured at home by mothers or birth attendants for non-facility births [26]. It would be practically challenging to suggest two different MUAC thresholds at birth i) to predict LBW and ii) to predict subsequent mortality. To identify higher risk infants, we recommend using birth MUAC<9.0cm, rather than using the *proxy of proxy* approach.

At the age of infant vaccinations, applying a MUAC threshold of <11.5cm among infants u6m would be consistent with current practice in children over 6 months (>6m) old but would identify a large proportion (17%) of the infants u6m for treatment. Importantly, below 6 months, the recommended nutritional intervention focuses on re-establishing exclusive breastfeeding [7]. Studies from therapeutic care centers have shown that this intervention is time consuming and labour-intensive and may be difficult to be effectively applied to a large group of infants [27–30]. Hence the need to target support to infants who may need it most. Our investigated MUAC thresholds between 11.5cm and 11.0cm would have identified 10.2% of the study population while MUAC<11.0cm identified 6.8%. We suggest applying <11.0cm as the primary definition for infants from 2–6 months most in need. Where resources allow an additional group between 11.0cm and 11.5cm can also be considered for lower intensity intervention.

The practical challenge in these recommendations is the introduction of a new MUAC cut-off among infants u6m which differs from that currently applied in children >6m. This challenge can be overcome by proper training of implementing health workers and understanding of the concepts of risks and the types and intensity of interventions.

We also found that weight<3.8kg (WAZ<-3 at age 2 months) was very strongly associated with mortality. As weight is most commonly measured at this age using routine road-to-health charts [31], our results suggest that it can be applied as an absolute measure (<3.8kg) to facilitate quick action. In early infancy, ‘small size due to LBW’ should not be considered an acceptable reason for not undertaking full assessment (e.g. of breastfeeding) and appropriate intervention as LBW is associated with increased risks of mortality.

### Missing anthropometry

This study had 59 infants with incalculable birth WLZ. This was strongly associated with mortality and is a major challenge in early identification of malnourished infants if applying WLZ [13, 32]. At two months of age, 147 infants defaulted visits and had missing anthropometry. Approximately 10% of these died during the 12 month follow-up, emphasizing that missing a scheduled follow up or clinic visit is an important indicator of risk and should not be ignored.

### Study strength and limitation

This study was a large longitudinal birth cohort in a resource-poor area with systematically collected data in the absence of specific intervention. Infants were enrolled at birth irrespective of anthropometric classification at birth. There was minimal loss to follow-up and mortality was accurately determined, although exact cause of death could not be ascertained.

### Conclusion

Among infants u6m, MUAC and weight thresholds are better discriminators of subsequent mortality than WLZ. At birth, where gestational age is unknown and weight is not possible to measure, birth MUAC can be applied to identify infants at risk. At the age of first vaccinations (approximately2 months), MUAC and WFA should be considered as the primary means of assessment to identify infants at high risk of mortality and thus intervention. Further research into the effectiveness, cost-effectiveness and coverage of using the proposed thresholds should be prioritized to further inform policy and practice.

## Supporting information

S1 Table Anthropometric summary of the participants at birth, two and twelve months. (PDF)

S2 Table Birth anthropometrics predictors of one-year mortality stratified by birth weight. (PDF)

S3 Table Birth anthropometrics predictors of one-year mortality excluding twins and premature infants (N = 1014). (PDF)

S4 Table Month two anthropometrics predictors of one-year mortality excluding twins and premature infants. (PDF)

S1 Fig Study participants flow chart. (PDF)

S2 Fig Nelson-Aalen cumulative hazard of one-year mortality stratified by birth MUAC<9.7 cm. (PDF)

S1 Dataset PLOSone_data.csv. (CSV)

## Abbreviations

LAZ: Length-for-age Z scores
LBW: Low birthweight
LMIC: Low and middle-income countries
MUAC: Mid-upper arm circumference
NBW: Normal Birth Weight
SAM: Severe Acute Malnutrition
u6m: Under 6 months
WAZ: Weight-for-age Z scores
WLZ: Weight-for-length Z score
