# Supplementary material for: Anthropometry at birth and at age of routine vaccination to predict mortality in the first year of life: A birth cohort study in BukinaFaso
Source: PLoS One. 2019 Mar 28;14(3):e0213523. doi: 10.1371/journal.pone.0213523 (PMC6438502; doi:10.1371/journal.pone.0213523)
Supplement: S1 Table — (PDF) [file pone.0213523.s001.pdf]

S1 Table: **Anthropometric summary of the participants at birth, two and twelve months.**

	At birth (N=1,103)	At month 2 (N=927)	At month 12 (N=941)
**All infants (N=1103)**			
Weight (Kg); mean ±sd	2.8 ± 0.5	4.9 ± 0.8	7.8 ± 1.1
MUAC in cm; mean ±sd	10.2 ± 1.1	12.6 ± 1.3	13.9 ± 1.5
Length (cm), mean ±sd	48.9 ± 2.6	56.4 ± 3.5	72.0 ± 2.8
WLZ; mean ±sd	-1.4 ± 1.6	-0.2 ± 2.0	-1.3 ± 1.3
WAZ; mean ±sd	-1.1 ± 1.1	-0.8 ± 1.3	-1.6 ± 1.2
LAZ; mean ±sd	-0.3 ± 1.4	-0.7 ± 1.7	-1.2 ± 1.2
ZHC; mean ±sd	-1.3 ± 1.5	-1.1 ± 1.8	-1.1 ± 1.3
**NBW infants (N=876)**	N=876	N=740	N=762
Weight (Kg); mean ±sd	3.0 ± 0.3	5.1 ± 0.7	8.0 ± 1.0
MUAC in cm; mean ±sd	10.5 ± 0.9	12.8 ± 1.2	14.0 ± 1.4
Length (cm), mean ±sd	49.5 ± 1.1	57.0 ± 3.2	72.4 ± 2.6
WLZ; mean ±sd	-1.1 ± 1.5	-0.04 ± 2.0	-1.2 ± 1.3
WAZ; mean ±sd	-0.7 ± 0.7	-0.5 ± 1.1	-1.4 ± 1.1
LAZ; mean ±sd	-0.1 ± 1.1	-0.4 ± 1.6	-1.1 ± 1.1
ZHC; mean ±sd	-1.1 ± 1.4	-0.9 ± 1.7	-1.0 ± 1.3
**LBW infants (N=227)**	N=227	N=187	N=179
Weight (Kg); mean ±sd	2.2 ± 0.3	4.0 ± 0.8	7.1 ± 1.0
MUAC in cm; mean ±sd	9.2 ± 0.9	11.6 ± 1.3	13.3 ± 1.4
Length (cm), mean ±sd	46.4 ± 2.8	54.1 ± 3.6	70.1 ± 2.9
WLZ; mean ±sd	-2.8 ± 1.2	-1.0 ± 1.9	-1.8 ± 1.3
WAZ; mean ±sd	-2.7 ± 0.8	-2.2 ± 1.4	-2.3 ± 1.2
LAZ; mean ±sd	-1.6 ± 1.5	-1.8 ± 1.8	-1.9 ± 1.2
ZHC; mean ±sd	-2.5 ± 1.6	-1.9 ± 2.0	-1.5 ± 1.2

MUAC-mid-upper arm circumference, WLZ-Weight-for-length z-score, WAZ-Weight-for-age z-score, LAZ-Length-for-age z-score, ZHC-Head circumference z-score, sd-Standard deviation
