# Supplementary material for: Anthropometry at birth and at age of routine vaccination to predict mortality in the first year of life: A birth cohort study in BukinaFaso
Source: PLoS One. 2019 Mar 28;14(3):e0213523. doi: 10.1371/journal.pone.0213523 (PMC6438502; doi:10.1371/journal.pone.0213523)
Supplement: S2 Table — (PDF) [file pone.0213523.s002.pdf]

S2 Table: **Birth anthropometrics predictors of one-year mortality stratified by birth weight.**

	LBW				NBW			
	**N (227)**	**Deaths N (%)**	**Adjusted HR (95% CI) #**	**P value**	**N (876)**	**Deaths N (%)**	**Adjusted HR (95% CI) #**	**P value**
**Birth anthropometrics (Deaths, N=86)**								
**Mid-upper arm circumference (MUAC-cm)**		N=38				N=48		
MUAC ≥10.0cm	61	9 (15)	Reference		715	42 (5.9)	Reference	
MUAC 9.0 to 10.0cm	104	12 (12)	1.20 (0.47, 3.07)	0.71	144	6 (4.2)	0.77 (0.32, 1.88)	0.57
MUAC<9.0cm	62	17 (27)	3.82 (1.61, 9.06)	0.002	17	0	-	
**Weight (kg)**								
Weight ≥2.5kg (WAZ ≥-2)	0	$			876	48 (5.5)		
Weight 2.0 to 2.5kg (WAZ -2 to -3)	179	26 (15)	-		-			
Weight<2.0kg (WAZ <-3)	48	12 (25)	-		-			
**Length (cm)**								
Length≥46.1cm (LAZ ≥-2)	128	17 (13)	Reference		827	45 (5.4)	Reference	
Length 44.2 to 46.1cm (LAZ -2 to -3)	54	8 (15)	0.99 (0.40, 2.49)	0.89	39	2 (5.1)	0.99 (0.23, 4.21)	0.98
Length<44.2cm (LAZ <-3)	45	13 (29)	1.85 (0.80, 4.30)	0.15	10	1 (10)	1.93 (0.24, 15.30)	0.54
**Weight-for-length z-score (WLZ)**								
WLZ≥-2	53	9 (17)	Reference		664	39 (5.9)	Reference	
WLZ -3 to -2	57	3 (5.3)	0.30 (0.08, 1.15)	0.08	124	4 (3.2)	0.52 (0.18, 1.48)	0.22
WLZ<-3	71	13 (18)	1.04 (0.44, 2.46)	0.92	75	4 (5.3)	0.92 (0.28, 2.97)	0.89
Missing WLZ	46	13 (28)	1.43 (0.55, 3.72)	0.46	13	1 (7.7)	1.38 (0.18, 10.73)	0.76
**Birth anthropometry (Continuous) AUCs**	N	Deaths						
MUAC (cm)	227	38	0.61 (0.50, 0.72)		876	48	0.43 (0.35, 0.51)	
Weight (kg)			-				-	
Length (cm)	227	38	0.64 (0.54, 0.74)		876	48	0.48 (0.40, 0.57)	
Weight-for-length z-score (WLZ)*	181	25	0.48 (0.35, 0.61)		863	47	0.47 (0.36,0.53)	

AUC-area under receiver operating characteristic, MUAC-mid-upper arm circumference, #-adjusted for gender, facility of birth and month of birth, *59 missing WLZ because their birth lengths<45cm, $-regression analysis for birth weight was not possible because the analysis is stratified by birth weight
