# Supplementary material for: Anthropometry at birth and at age of routine vaccination to predict mortality in the first year of life: A birth cohort study in BukinaFaso
Source: PLoS One. 2019 Mar 28;14(3):e0213523. doi: 10.1371/journal.pone.0213523 (PMC6438502; doi:10.1371/journal.pone.0213523)
Supplement: S3 Table — (PDF) [file pone.0213523.s003.pdf]

S3 Table: **Birth anthropometrics predictors of one-year mortality excluding twins and premature (N=1014).**

			Crude (95% CI)		Adjusted (95% CI)[#]	
	**N (1014)**	**Deaths N (%)**	**HR (95% CI)**	**P value**	**HR (95% CI)**	**P value**
**Birth anthropometrics (Deaths, N=86)**						
**Mid-upper arm circumference (MUAC-cm)**						
MUAC ≥10.0cm	763	48 (6.3)	Reference		Reference	
MUAC 9.0 to 10.0cm	213	16 (7.5)	1.21 (0.69, 2.13)	0.51	1.41 (0.78, 2.53)	0.26
MUAC<9.0cm	38	2 (5.3)	0.85 (0.21, 3.48)	0.82	1.03 (0.25, 4.31)	0.97
**Weight (kg)**						
Weight ≥2.5kg (WAZ ≥-2)	853	47 (5.5)	Reference		Reference	
Weight 2.0 to 2.5kg (WAZ -2 to -3)	148	18 (12)	2.23 (1.29, 3.84)	0.004	2.32 (1.34, 4.03)	0.003
Weight<2.0kg (WAZ <-3)	13	1 (7.7)	1.50 (0.21, 10.91)	0.69	1.39 (0.19, 10.17)	0.75
**Length (cm)**						
Length≥46.1cm (LAZ ≥-2)	913	58 (6.4)	Reference		Reference	
Length 44.2 to 46.1cm (LAZ -2 to -3)	75	5 (6.7)	1.06 (0.43, 2.65)	0.89	1.06 (0.42, 2.67)	0.91
Length<44.2cm (LAZ <-3)	26	3 (12)	1.95 (0.61, 6.22)	0.26	2.10 (0.65, 6.81)	0.22
**Weight-for-length z-score (WLZ)**						
WLZ≥-2	691	44 (6.4)	Reference		Reference	
WLZ -3 to -2	171	7 (4.1)	0.62 (0.28, 1.38)	0.24	0.63 (0.28, 1.42)	0.27
WLZ<-3	122	12 (9.8)	1.54 (0.81, 2.91)	0.19	1.64 (0.83, 3.22)	0.15
Missing WLZ	30	3 (10)	1.64 (0.51, 5.29)	0.41	1.83 (0.56, 5.98)	0.32
**Birth anthropometry (Continuous) AUCs**	N	Deaths				
MUAC (cm)	1014	66	0.51 (0.44, 0.58)			
Weight (kg)	1014	66	0.52 (0.44, 0.60)			
Length (cm)	1014	66	0.53 (0.46, 0.61)			
Weight-for-length z-score (WLZ)*	984	63	0.50 (0.42, 0.58)			

AUC-area under receiver operating characteristic, MUAC-mid-upper arm circumference, #-adjusted for gender, facility of birth and month of birth, *30 missing WLZ because their birth lengths<45cm.
