# Supplementary material for: Anthropometry at birth and at age of routine vaccination to predict mortality in the first year of life: A birth cohort study in BukinaFaso
Source: PLoS One. 2019 Mar 28;14(3):e0213523. doi: 10.1371/journal.pone.0213523 (PMC6438502; doi:10.1371/journal.pone.0213523)
Supplement: S4 Table — (PDF) [file pone.0213523.s004.pdf]

S4 Table: **Month two anthropometrics predictors of one-year mortality excluding twins and premature.**

			Crude (95% CI)		Adjusted (95% CI)[#]	
	N (987)	**Deaths N (%)**	HR (95% CI)	P value	**HR (95% CI)**	P value
**Month 2 Anthropometry (Death, N=49)**						
**Mid-upper arm circumference (MUAC-cm)**						
MUAC ≥11.5cm	731	24 (3.3)	Reference		Reference	
MUAC 11.0 to 11.5cm	78	6 (7.7)	2.50 (1.02, 6.11)	0.04	2.85 (1.14, 7.13)	0.03
MUAC<11.0cm	40	4 (10)	3.14 (1.09, 9.05)	0.03	3.86 (1.31, 11.38)	0.01
Missing MUAC	138	12 (8.7)	2.85 (1.43, 5.70)	0.003	2.62 (1.27, 5.39)	0.009
**Weight (kg)**						
Weight≥4.3kg (WAZ≥-2)	707	24 (3.4)	Reference		Reference	
Weight 3.8 to 4.3kg (WAZ -2 to -3)	94	4 (4.3)	1.29 (0.45, 3.71)	0.64	1.29 (0.44, 3.76)	0.64
Weight<3.8kg (WAZ<-3)	48	6 (13)	4.01 (1.64, 9.80)	0.002	4.18 (1.68, 10.38)	0.002
Missing weight	138	12 (8.7)	2.76 (1.38, 5.52)	0.004	2.54 (1.23, 5.22)	0.01
**Length (cm)**						
Length≥54.4cm (LAZ≥-2)	629	23 (3.7)	Reference		Reference	
Length 52.4 to 54.4cm (HAZ -3 to -2)	131	5 (3.8)	1.06 (0.40, 2.78)	0.91	1.14 (0.43, 3.08)	0.79
Length<52.4cm (HAZ <-3)	89	6 (6.7)	1.95 (0.79, 4.79)	0.15	1.81 (0.69, 4.76)	0.23
Missing length	138	12 (8.7)	2.56 (1.27, 5.15)	0.008	2.44 (1.16, 5.18)	0.02
**Weight-for-length z-score (WLZ)**						
WLZ≥-2	715	26 (3.6)	Reference		Reference	
WLZ -3 to -2	80	4 (5.0)	1.40 (0.49, 4.02)	0.53	1.33 (0.46, 3.88)	0.60
WLZ<-3*	53	4 (7.6)	2.10 (0.73, 6.03)	0.17	1.89 (0.65, 5.49)	0.24
Missing WLZ	139	12 (8.6)	2.54 (1.28, 5.04)	0.008	2.31 (1.13, 4.70)	0.02
**Month 2 anthropometry (Continuous) AUCs**	N	Deaths				
MUAC (cm)	849	34	0.56 (0.45, 0.67)			
Weight (kg)	849	34	0.58 (0.48, 0.69)			
Length (cm)	849	34	0.56 (0.46, 0.67)			
WLZ	849	34	0.54 (0.43, 0.66)			
**Concurrent Undernutrition**						
No stunting, wasting or underweight	555	19 (3.4)	Reference		Reference	
Concurrent stunted & wasted	5	1 (20)	5.92 (0.79, 44.29)	0.08	5.06 (0.64, 40.14)	0.13
Concurrent wasted & underweight	38	3 (8.0)	2.41 (0.71, 8.15)	0.16	2.21 (0.62, 7.85)	0.22
Concurrent stunted & underweight	54	5 (9.3)	2.98 (1.11, 7.99)	0.03	2.33 (0.82, 6.59)	0.11
**Low birth weight status at birth**						
Birth weight ≥2.5kg	833	33 (4.0)	Reference		Reference	
Birth weight <2.5kg	154	13 (4.4)	2.12 (1.12, 4.03)	0.02	2.14 (1.12, 4.10)	0.02

WAZ-Weight for age z-score, LAZ-Length for age z-score, WLZ- Weight for length z-scores, AUC-area under receiver operating characteristic, #-adjusted for gender, facility of birth and month of birth. *1 missing WLZ because their month 2 lengths<45cm.
