# Supplementary material for: Anthropometry at birth and at age of routine vaccination to predict mortality in the first year of life: A birth cohort study in BukinaFaso
Source: PLoS One. 2019 Mar 28;14(3):e0213523. doi: 10.1371/journal.pone.0213523 (PMC6438502; doi:10.1371/journal.pone.0213523)
Supplement: S1 Fig — (PDF) [file pone.0213523.s005.pdf]

S1 Figure: **Study participants flow chart**.

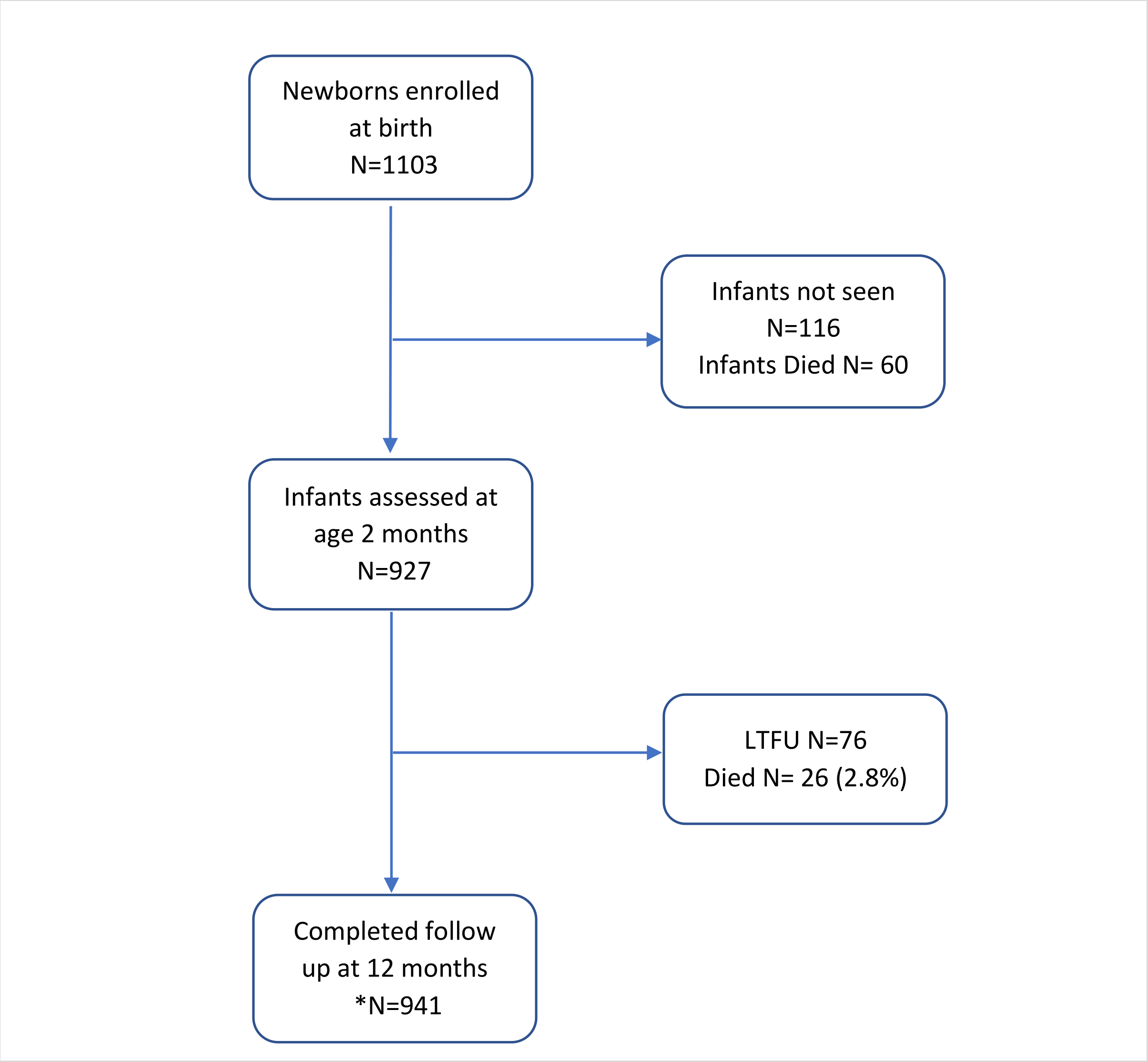

LTFU-lost-to-follow-up (Infants whose outcome not assessed in last visit). * N includes Infants not seen at the month 2 visit but seen in the last visit).
