# Supplementary material for: Anthropometry at birth and at age of routine vaccination to predict mortality in the first year of life: A birth cohort study in BukinaFaso
Source: PLoS One. 2019 Mar 28;14(3):e0213523. doi: 10.1371/journal.pone.0213523 (PMC6438502; doi:10.1371/journal.pone.0213523)
Supplement: S2 Fig — (PDF) [file pone.0213523.s006.pdf]

S2 Figure: **Nelson-Aalen cumulative hazard of one-year mortality stratified by birth MUAC<9.7 cm.**

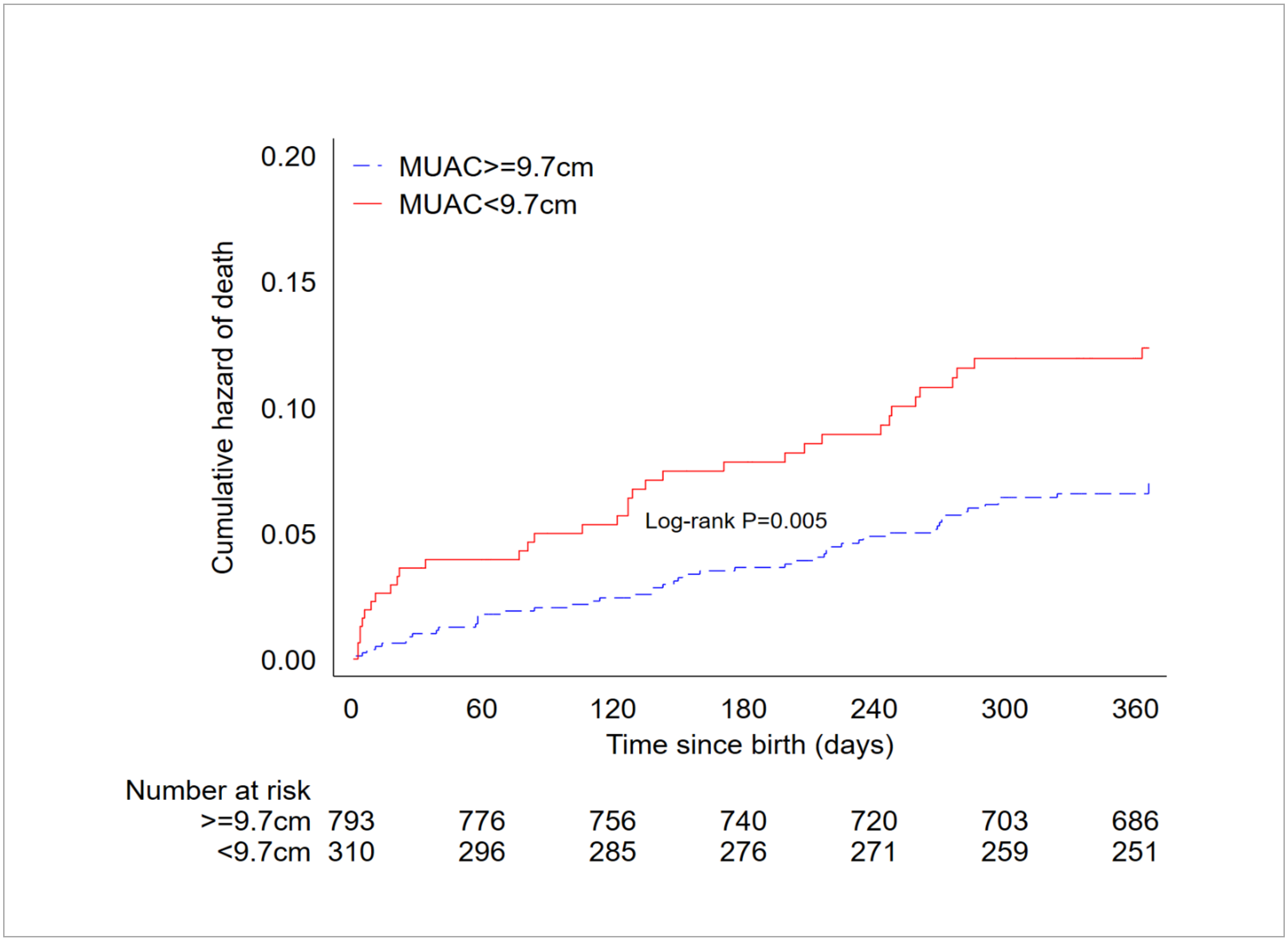


MUAC-mid-upper arm circumference.
